# Crystal structure of (*E*)-1-([1,1′-biphen­yl]-4-yl)-3-(3-nitro­phen­yl)prop-2-en-1-one

**DOI:** 10.1107/S2056989015000523

**Published:** 2015-01-17

**Authors:** D. Shanthi, T. Vidhyasagar, K. Rajeswari, M. Kayalvizhi, G. Vasuki, A. Thiruvalluvar

**Affiliations:** a Department of Chemistry, Annamalai University, Annamalai Nagar 608 002, Tamilnadu, India; b Department of Physics, Kunthavai Naachiar Government Arts College (W) (Autonomous), Thanjavur 613 007, Tamilnadu, India; c Postgraduate Research Department of Physics, Rajah Serfoji Government College (Autonomous), Thanjavur 613 005, Tamilnadu, India

**Keywords:** crystal structure, chalcones, C—H⋯π inter­actions

## Abstract

In the title compound, C_21_H_15_NO_3_, the mol­ecule has an *E* conformation about the C=C bond, and the C—C=C—C torsion angle is −178.24 (18)°. In the mol­ecule, the planes of the terminal rings are twisted by an angle of 42.19 (10)°, while the biphenyl part is not planar, with a dihedral angle between the rings of 39.2 (1)°. The dihedral angle between the nitro­phenyl ring and the inner benzene ring is 5.56 (9)°. The 3-nitro group is approximately coplanar with the benzene ring to which it is attached [O—N—C—C = 0.1 (3)°]. In the crystal, mol­ecules are linked *via* C—H⋯π inter­actions, involving the terminal benzene rings, forming corrugated layers parallel to (100).

## Related literature   

For the biological activities of chalcones, see: Nowakowska (2007 ▸); Liu *et al.* (2008 ▸); Wu *et al.* (2010 ▸); Singh *et al.* (2012 ▸). For non-linear optical (NLO) properties of chalcone derivatives, see: Uchida *et al.* (1998 ▸); Indira *et al.* (2002 ▸). For the crystal structures of related compounds, see: Shanthi *et al.* (2014 ▸); Vidhyasagar *et al.* (2015*a* ▸,*b* ▸).
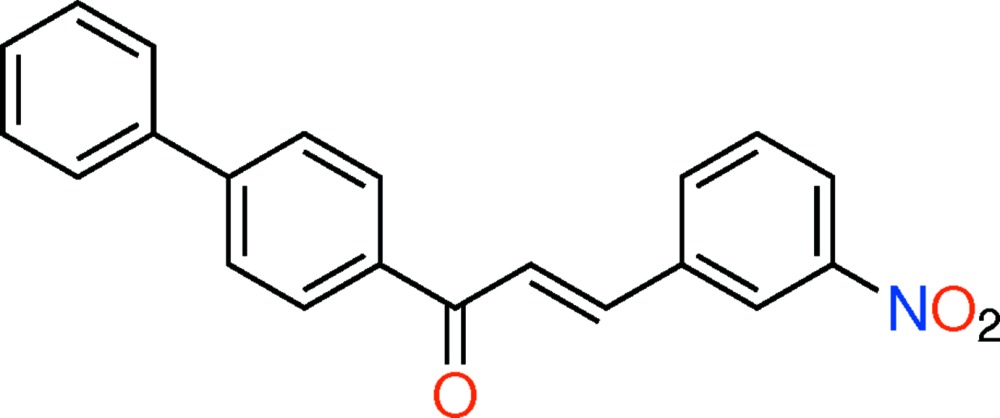


## Experimental   

### Crystal data  

C_21_H_15_NO_3_
*M* *_r_* = 329.34
Monoclinic, 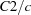

*a* = 17.6546 (5) Å
*b* = 6.1464 (2) Å
*c* = 30.0234 (9) Å
β = 99.899 (4)°
*V* = 3209.40 (17) Å^3^
*Z* = 8
Mo *K*α radiation
μ = 0.09 mm^−1^
*T* = 296 K
0.35 × 0.35 × 0.30 mm

### Data collection  

Bruker Kappa APEXII CCD diffractometer
Absorption correction: multi-scan (*SADABS*; Bruker, 2004 ▸) *T*_min_ = 0.691, *T*_max_ = 0.745
26025 measured reflections
3170 independent reflections
2500 reflections with *I* > 2σ(*I*)
*R*_int_ = 0.026

### Refinement  

*R*[*F* ^2^ > 2σ(*F* ^2^)] = 0.049
*wR*(*F* ^2^) = 0.138
*S* = 1.08
3170 reflections
226 parameters
H-atom parameters constrained
Δρ_max_ = 0.26 e Å^−3^
Δρ_min_ = −0.17 e Å^−3^

### 

Data collection: *APEX2* (Bruker, 2004 ▸); cell refinement: *APEX2* and *SAINT* (Bruker, 2004 ▸); data reduction: *SAINT* and *XPREP* (Bruker, 2004 ▸); program(s) used to solve structure: *SIR92* (Altomare *et al.*, 1994 ▸); program(s) used to refine structure: *SHELXL2014* (Sheldrick, 2015 ▸); molecular graphics: *ORTEP-3 for Windows* (Farrugia, 2012 ▸) and *Mercury* (Macrae *et al.*, 2008 ▸); software used to prepare material for publication: *SHELXL2014*, *PLATON* (Spek, 2009 ▸) and *publCIF* (Westrip, 2010 ▸).

## Supplementary Material

Crystal structure: contains datablock(s) global, I. DOI: 10.1107/S2056989015000523/su5060sup1.cif

Structure factors: contains datablock(s) I. DOI: 10.1107/S2056989015000523/su5060Isup2.hkl

Supporting information file. DOI: 10.1107/S2056989015000523/su5060Isup3.cdx

Supporting information file. DOI: 10.1107/S2056989015000523/su5060Isup4.cml

**Fig. 1.**
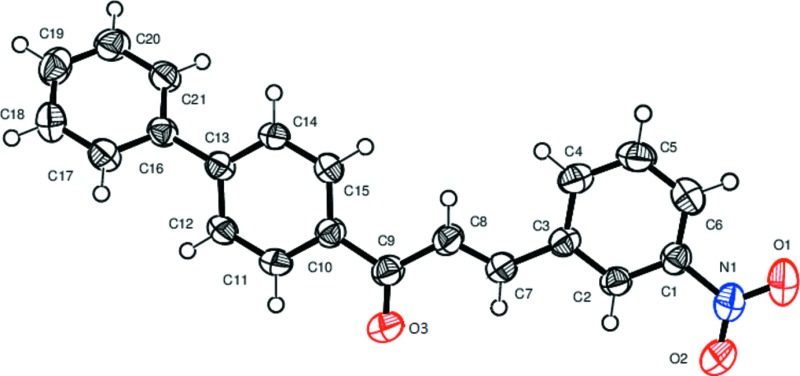
The molecular structure of the title compound, with atom labelling. Displacement ellipsoids are drawn at the 50% probability level.

**Fig. 2.**
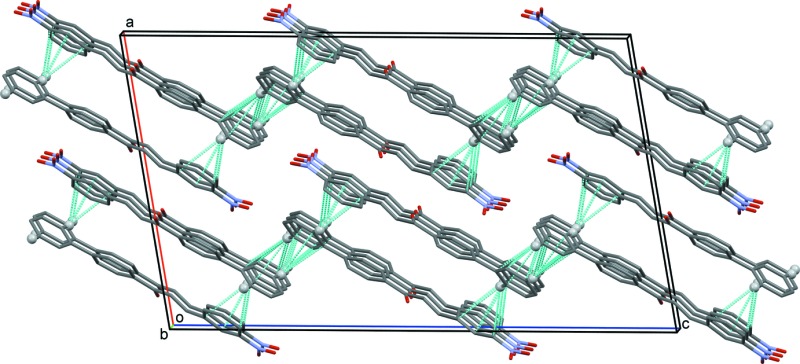
A view along the b axis of the crystal packing of the title compound. The C-H···π interactions are shown as dashed lines (see Table 1 for details; for clarity only the H atoms participating in these interactions are shown).

. DOI: 10.1107/S2056989015000523/su5060fig1.tif
The mol­ecular structure of the title compound, with atom labelling. Displacement ellipsoids are drawn at the 50% probability level.

b . DOI: 10.1107/S2056989015000523/su5060fig2.tif
A view along the *b* axis of the crystal packing of the title compound. The C-H⋯π inter­actions are shown as dashed lines (see Table 1 for details; for clarity only the H atoms participating in these inter­actions are shown).

CCDC reference: 991338

Additional supporting information:  crystallographic information; 3D view; checkCIF report

## Figures and Tables

**Table 1:** Hydrogen-bond geometry (, ) *Cg*1 and *Cg*3 are the centroids of the nitrobenzene ring C1C6 and the phenyl ring C16C21, respectively.

*D*H*A*	*D*H	H*A*	*D* *A*	*D*H*A*
C17H17*Cg*1^i^	0.93	2.93	3.637(2)	133
C20H20*Cg*3^ii^	0.93	2.90	3.565(2)	129

Symmetry codes: (i) 
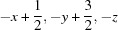
; (ii) 
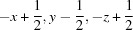
.

## supplementary crystallographic information

### S1. Comment

Chalcones and their analogs have been used for a wide range of biological activities, including antimicrobial, antitumor, anti-inflammatory, antifungal and antioxidant activities (Nowakowska, 2007; Liu *et al.* 2008; Wu *et al.* 2010; Singh *et al.* 2012). Chalcone derivatives also show considerable promise as organic non-linear optical materials (Uchida *et al.*, 1998; Indira *et al.*, (2002). The crystal structures of related compounds have been reported (Shanthi *et al.*, 2014; Vidhyasagar *et al.*, 2015*a*,b). As part of our on-going research on biphenyl chalcone derivatives, the title compound was synthesized and its crystal structure is reported on herein.

In the title compound, Fig. 1, the molecule exists as an *E* conformer with the C3—C7—C8—C9 torsion angle being -178.24 (18)°. The terminal rings (C1—C6) and (C16—C21) are twisted by an angle of 42.19 (10)°, while the biphenyl (C10—C15 and C16—C21) part is not planar, the dihedral angle between the planes of these rings being 39.2 (1)°. The dihedral angle between the benzene rings (C1—C6) and (C10—C15) is 5.56 (9)°. The 3-nitro group is approximately coplanar with the benzene ring to which it is attached [O2—N1—C1—C2 = 0.1 (3)°].

In the crystal, molecules are linked *via* C-H···π interactions, involving the terminal benzene rings (C1—C6) and (C16—C21), forming corrugated layers parallel to (100); see Table 1 and Fig. 2.

### S2. Experimental

A mixture of 4-acetylbiphenyl (1.96 g, 10 mmol) and 3-nitro benzaldehyde (1.07 g, 10 mmol) in ethanol (25 ml) in the presence of NaOH (10 ml 30%) were heated in a water bath for 30 min. and then allowed to cool. The solid that separated was filtered and recrystallized from ethanol. The yellow crystals of the title compound used for the X-ray diffraction study were grown by slow evaporation from acetone (yield: 2.5 g; 75%).

### S3. Refinement

All H-atoms were positioned geometrically and allowed to ride on their parent atoms: C—H = 0.93 Å with *U*_iso_(H) = 1.2 *U*_eq_(C).

### Crystal data

**Table:**

C_21_H_15_NO_3_	*F*(000) = 1376
*M**_r_* = 329.34	*D*_x_ = 1.363 Mg m^−^^3^
Monoclinic, *C*2/*c*	Melting point: 462.9 K
Hall symbol: -C 2yc	Mo *K*α radiation, λ = 0.71073 Å
*a* = 17.6546 (5) Å	Cell parameters from 9326 reflections
*b* = 6.1464 (2) Å	θ = 2.5–15.9°
*c* = 30.0234 (9) Å	µ = 0.09 mm^−^^1^
β = 99.899 (4)°	*T* = 296 K
*V* = 3209.40 (17) Å^3^	Block, yellow
*Z* = 8	0.35 × 0.35 × 0.30 mm

### Data collection

**Table:**

Bruker Kappa APEXII CCD diffractometer	3170 independent reflections
Radiation source: fine-focus sealed tube	2500 reflections with *I* > 2σ(*I*)
Graphite monochromator	*R*_int_ = 0.026
ω and φ scan	θ_max_ = 26.1°, θ_min_ = 2.3°
Absorption correction: multi-scan (*SADABS*; Bruker, 2004)	*h* = −21→21
*T*_min_ = 0.691, *T*_max_ = 0.745	*k* = −7→7
26025 measured reflections	*l* = −37→36

### Refinement

**Table:**

Refinement on *F*^2^	Primary atom site location: structure-invariant direct methods
Least-squares matrix: full	Secondary atom site location: difference Fourier map
*R*[*F*^2^ > 2σ(*F*^2^)] = 0.049	Hydrogen site location: inferred from neighbouring sites
*wR*(*F*^2^) = 0.138	H-atom parameters constrained
*S* = 1.08	*w* = 1/[σ^2^(*F*_o_^2^) + (0.0541*P*)^2^ + 3.3754*P*] where *P* = (*F*_o_^2^ + 2*F*_c_^2^)/3
3170 reflections	(Δ/σ)_max_ < 0.001
226 parameters	Δρ_max_ = 0.26 e Å^−^^3^
0 restraints	Δρ_min_ = −0.17 e Å^−^^3^

### Special details

**Table:**

Geometry. Bond distances, angles *etc*. have been calculated using the rounded fractional coordinates. All su's are estimated from the variances of the (full) variance-covariance matrix. The cell e.s.d.'s are taken into account in the estimation of distances, angles and torsion angles

### Fractional atomic coordinates and isotropic or equivalent isotropic displacement parameters (Å^2^)

**Table:**

	*x*	*y*	*z*	*U*_iso_*/*U*_eq_	
O1	0.57652 (12)	0.4252 (3)	−0.19910 (6)	0.0883 (8)	
O2	0.58837 (11)	0.7332 (3)	−0.16537 (6)	0.0773 (7)	
O3	0.39281 (11)	1.0140 (3)	0.01671 (6)	0.0693 (6)	
N1	0.56468 (10)	0.5469 (3)	−0.16914 (6)	0.0562 (7)	
C1	0.51892 (10)	0.4648 (3)	−0.13607 (6)	0.0420 (6)	
C2	0.50490 (10)	0.6028 (3)	−0.10237 (6)	0.0407 (6)	
C3	0.45859 (11)	0.5329 (3)	−0.07197 (6)	0.0404 (6)	
C4	0.43071 (12)	0.3208 (3)	−0.07611 (7)	0.0486 (7)	
C5	0.44639 (12)	0.1864 (3)	−0.10995 (8)	0.0544 (7)	
C6	0.49053 (12)	0.2569 (4)	−0.14085 (7)	0.0506 (7)	
C7	0.43910 (11)	0.6866 (3)	−0.03818 (6)	0.0458 (6)	
C8	0.38646 (11)	0.6597 (3)	−0.01229 (7)	0.0475 (7)	
C9	0.37029 (11)	0.8276 (3)	0.01956 (6)	0.0454 (6)	
C10	0.32643 (10)	0.7680 (3)	0.05601 (6)	0.0398 (6)	
C11	0.31668 (12)	0.9253 (3)	0.08779 (7)	0.0487 (7)	
C12	0.27956 (12)	0.8788 (3)	0.12315 (7)	0.0485 (7)	
C13	0.25004 (11)	0.6723 (3)	0.12863 (6)	0.0386 (5)	
C14	0.25952 (11)	0.5155 (3)	0.09672 (6)	0.0422 (6)	
C15	0.29722 (11)	0.5609 (3)	0.06115 (6)	0.0433 (6)	
C16	0.21041 (11)	0.6233 (3)	0.16715 (6)	0.0397 (6)	
C17	0.16140 (11)	0.7741 (3)	0.18176 (7)	0.0470 (6)	
C18	0.12348 (13)	0.7254 (4)	0.21715 (7)	0.0570 (8)	
C19	0.13376 (13)	0.5265 (4)	0.23841 (7)	0.0586 (8)	
C20	0.18328 (13)	0.3770 (4)	0.22494 (7)	0.0545 (7)	
C21	0.22157 (12)	0.4246 (3)	0.18959 (6)	0.0461 (6)	
H2	0.52615	0.74161	−0.09987	0.0488*	
H4	0.40097	0.26912	−0.05564	0.0582*	
H5	0.42694	0.04543	−0.11206	0.0653*	
H6	0.50062	0.16684	−0.16403	0.0607*	
H7	0.46667	0.81623	−0.03468	0.0549*	
H8	0.35878	0.53030	−0.01430	0.0570*	
H11	0.33570	1.06482	0.08500	0.0584*	
H12	0.27398	0.98736	0.14392	0.0582*	
H14	0.23999	0.37647	0.09938	0.0506*	
H15	0.30313	0.45231	0.04045	0.0520*	
H17	0.15406	0.90890	0.16762	0.0564*	
H18	0.09084	0.82768	0.22665	0.0683*	
H19	0.10733	0.49326	0.26179	0.0703*	
H20	0.19103	0.24360	0.23963	0.0654*	
H21	0.25507	0.32293	0.18074	0.0554*	

### Atomic displacement parameters (Å^2^)

**Table:**

	*U*^11^	*U*^22^	*U*^33^	*U*^12^	*U*^13^	*U*^23^
O1	0.1143 (15)	0.0909 (14)	0.0732 (12)	−0.0074 (12)	0.0544 (11)	−0.0205 (11)
O2	0.0927 (13)	0.0690 (12)	0.0825 (12)	−0.0205 (10)	0.0494 (10)	−0.0031 (10)
O3	0.0946 (12)	0.0492 (9)	0.0733 (11)	−0.0190 (9)	0.0408 (10)	−0.0026 (8)
N1	0.0556 (11)	0.0650 (13)	0.0523 (10)	0.0023 (10)	0.0215 (9)	−0.0020 (9)
C1	0.0377 (10)	0.0457 (11)	0.0434 (10)	0.0031 (8)	0.0092 (8)	0.0021 (8)
C2	0.0402 (10)	0.0382 (10)	0.0442 (10)	−0.0031 (8)	0.0088 (8)	0.0019 (8)
C3	0.0422 (10)	0.0416 (10)	0.0370 (9)	−0.0048 (8)	0.0056 (8)	0.0031 (8)
C4	0.0500 (11)	0.0459 (12)	0.0503 (11)	−0.0088 (9)	0.0102 (9)	0.0054 (9)
C5	0.0563 (13)	0.0384 (11)	0.0686 (14)	−0.0081 (10)	0.0107 (10)	−0.0008 (10)
C6	0.0488 (11)	0.0497 (12)	0.0527 (12)	0.0032 (10)	0.0067 (9)	−0.0095 (10)
C7	0.0531 (12)	0.0428 (11)	0.0431 (10)	−0.0056 (9)	0.0132 (9)	−0.0008 (9)
C8	0.0507 (11)	0.0485 (12)	0.0462 (11)	−0.0119 (9)	0.0168 (9)	−0.0038 (9)
C9	0.0480 (11)	0.0453 (11)	0.0434 (10)	−0.0076 (9)	0.0092 (8)	−0.0002 (9)
C10	0.0417 (10)	0.0395 (10)	0.0383 (9)	−0.0011 (8)	0.0074 (8)	0.0017 (8)
C11	0.0619 (13)	0.0327 (10)	0.0537 (12)	−0.0081 (9)	0.0166 (10)	−0.0037 (9)
C12	0.0684 (13)	0.0342 (10)	0.0460 (11)	−0.0029 (9)	0.0183 (10)	−0.0070 (9)
C13	0.0440 (10)	0.0342 (9)	0.0369 (9)	0.0023 (8)	0.0051 (8)	−0.0004 (8)
C14	0.0531 (11)	0.0316 (9)	0.0432 (10)	−0.0051 (8)	0.0124 (9)	−0.0014 (8)
C15	0.0519 (11)	0.0384 (10)	0.0414 (10)	−0.0036 (9)	0.0129 (8)	−0.0072 (8)
C16	0.0457 (10)	0.0374 (10)	0.0353 (9)	−0.0015 (8)	0.0049 (8)	−0.0034 (8)
C17	0.0542 (12)	0.0417 (11)	0.0446 (10)	0.0055 (9)	0.0069 (9)	−0.0014 (9)
C18	0.0578 (13)	0.0665 (15)	0.0493 (12)	0.0100 (11)	0.0170 (10)	−0.0055 (11)
C19	0.0648 (14)	0.0724 (16)	0.0415 (11)	−0.0057 (12)	0.0175 (10)	−0.0007 (11)
C20	0.0713 (14)	0.0500 (12)	0.0424 (11)	−0.0045 (11)	0.0107 (10)	0.0051 (9)
C21	0.0580 (12)	0.0384 (10)	0.0423 (10)	0.0015 (9)	0.0095 (9)	−0.0021 (8)

### Geometric parameters (Å, º)

**Table:**

O1—N1	1.215 (3)	C16—C17	1.390 (3)
O2—N1	1.218 (3)	C16—C21	1.392 (3)
O3—C9	1.221 (3)	C17—C18	1.383 (3)
N1—C1	1.473 (3)	C18—C19	1.377 (3)
C1—C2	1.375 (3)	C19—C20	1.376 (3)
C1—C6	1.371 (3)	C20—C21	1.384 (3)
C2—C3	1.394 (3)	C2—H2	0.9300
C3—C4	1.392 (3)	C4—H4	0.9300
C3—C7	1.470 (3)	C5—H5	0.9300
C4—C5	1.374 (3)	C6—H6	0.9300
C5—C6	1.380 (3)	C7—H7	0.9300
C7—C8	1.320 (3)	C8—H8	0.9300
C8—C9	1.468 (3)	C11—H11	0.9300
C9—C10	1.491 (3)	C12—H12	0.9300
C10—C11	1.390 (3)	C14—H14	0.9300
C10—C15	1.392 (3)	C15—H15	0.9300
C11—C12	1.370 (3)	C17—H17	0.9300
C12—C13	1.393 (3)	C18—H18	0.9300
C13—C14	1.389 (3)	C19—H19	0.9300
C13—C16	1.482 (3)	C20—H20	0.9300
C14—C15	1.381 (3)	C21—H21	0.9300
			
O1—N1—O2	123.20 (19)	C18—C19—C20	119.8 (2)
O1—N1—C1	118.18 (18)	C19—C20—C21	120.2 (2)
O2—N1—C1	118.62 (17)	C16—C21—C20	120.62 (19)
N1—C1—C2	118.28 (16)	C1—C2—H2	120.00
N1—C1—C6	118.80 (17)	C3—C2—H2	120.00
C2—C1—C6	122.90 (18)	C3—C4—H4	119.00
C1—C2—C3	119.46 (17)	C5—C4—H4	119.00
C2—C3—C4	117.94 (17)	C4—C5—H5	119.00
C2—C3—C7	119.16 (17)	C6—C5—H5	119.00
C4—C3—C7	122.87 (17)	C1—C6—H6	121.00
C3—C4—C5	121.08 (19)	C5—C6—H6	121.00
C4—C5—C6	121.14 (19)	C3—C7—H7	117.00
C1—C6—C5	117.44 (19)	C8—C7—H7	117.00
C3—C7—C8	126.72 (18)	C7—C8—H8	119.00
C7—C8—C9	122.06 (17)	C9—C8—H8	119.00
O3—C9—C8	120.80 (18)	C10—C11—H11	119.00
O3—C9—C10	119.91 (18)	C12—C11—H11	119.00
C8—C9—C10	119.28 (16)	C11—C12—H12	119.00
C9—C10—C11	118.31 (17)	C13—C12—H12	119.00
C9—C10—C15	123.72 (16)	C13—C14—H14	119.00
C11—C10—C15	117.91 (17)	C15—C14—H14	119.00
C10—C11—C12	121.27 (17)	C10—C15—H15	120.00
C11—C12—C13	121.35 (18)	C14—C15—H15	120.00
C12—C13—C14	117.32 (17)	C16—C17—H17	120.00
C12—C13—C16	120.93 (17)	C18—C17—H17	120.00
C14—C13—C16	121.75 (17)	C17—C18—H18	120.00
C13—C14—C15	121.64 (17)	C19—C18—H18	120.00
C10—C15—C14	120.51 (17)	C18—C19—H19	120.00
C13—C16—C17	120.93 (17)	C20—C19—H19	120.00
C13—C16—C21	120.62 (17)	C19—C20—H20	120.00
C17—C16—C21	118.45 (17)	C21—C20—H20	120.00
C16—C17—C18	120.55 (19)	C16—C21—H21	120.00
C17—C18—C19	120.4 (2)	C20—C21—H21	120.00
			
O1—N1—C1—C2	−179.46 (19)	C9—C10—C11—C12	−177.45 (19)
O2—N1—C1—C2	0.1 (3)	C15—C10—C11—C12	0.0 (3)
O1—N1—C1—C6	−1.1 (3)	C9—C10—C15—C14	177.65 (18)
O2—N1—C1—C6	178.40 (19)	C11—C10—C15—C14	0.3 (3)
N1—C1—C2—C3	176.84 (17)	C10—C11—C12—C13	−0.1 (3)
C6—C1—C2—C3	−1.4 (3)	C11—C12—C13—C14	−0.2 (3)
N1—C1—C6—C5	−178.54 (18)	C11—C12—C13—C16	179.62 (19)
C2—C1—C6—C5	−0.3 (3)	C12—C13—C14—C15	0.6 (3)
C1—C2—C3—C4	2.5 (3)	C16—C13—C14—C15	−179.26 (18)
C1—C2—C3—C7	−175.45 (17)	C12—C13—C16—C17	39.4 (3)
C2—C3—C4—C5	−1.9 (3)	C12—C13—C16—C21	−140.6 (2)
C7—C3—C4—C5	175.90 (19)	C14—C13—C16—C17	−140.8 (2)
C2—C3—C7—C8	167.81 (19)	C14—C13—C16—C21	39.3 (3)
C4—C3—C7—C8	−10.0 (3)	C13—C14—C15—C10	−0.6 (3)
C3—C4—C5—C6	0.3 (3)	C13—C16—C17—C18	178.72 (19)
C4—C5—C6—C1	0.9 (3)	C21—C16—C17—C18	−1.4 (3)
C3—C7—C8—C9	−178.24 (18)	C13—C16—C21—C20	−178.60 (19)
C7—C8—C9—O3	15.5 (3)	C17—C16—C21—C20	1.5 (3)
C7—C8—C9—C10	−163.88 (18)	C16—C17—C18—C19	0.0 (3)
O3—C9—C10—C11	−4.1 (3)	C17—C18—C19—C20	1.3 (3)
O3—C9—C10—C15	178.56 (19)	C18—C19—C20—C21	−1.2 (3)
C8—C9—C10—C11	175.31 (18)	C19—C20—C21—C16	−0.2 (3)
C8—C9—C10—C15	−2.0 (3)		

### Hydrogen-bond geometry (Å, º)

Cg1 and Cg3 are the centroids of the nitrobenzene ring C1–C6 and the phenyl ring C16–C21, respectively.

**Table:**

*D*—H···*A*	*D*—H	H···*A*	*D*···*A*	*D*—H···*A*
C17—H17···*Cg*1^i^	0.93	2.93	3.637 (2)	133
C20—H20···*Cg*3^ii^	0.93	2.90	3.565 (2)	129

Symmetry codes: (i) −*x*+1/2, −*y*+3/2, −*z*; (ii) −*x*+1/2, *y*−1/2, −*z*+1/2.
