# Supplementary figures and images for: Crystal structure of (E)-1-([1,1′-biphen­yl]-4-yl)-3-(3-nitro­phen­yl)prop-2-en-1-one
Source: Acta Crystallogr E Crystallogr Commun. 2015 Jan 17;71(Pt 2):o119–20. doi: 10.1107/S2056989015000523 (PMC4384577; doi:10.1107/S2056989015000523)

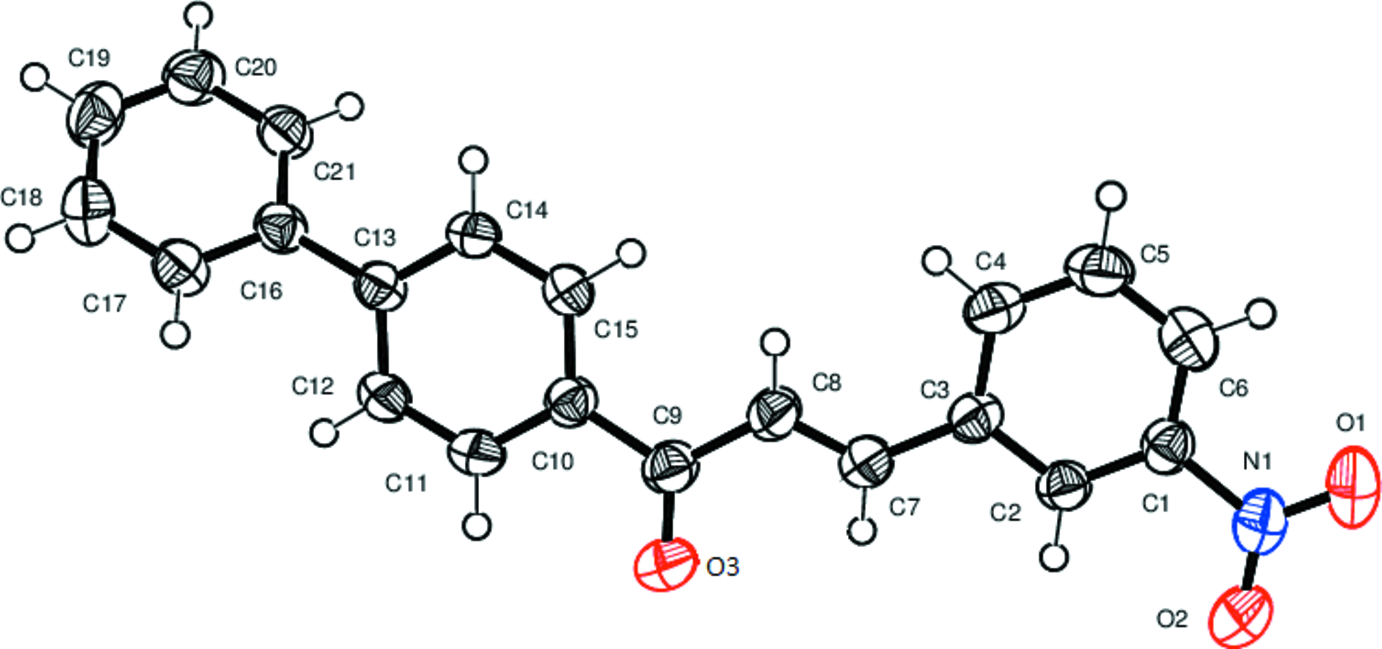

Supplement: Supplementary file 5 [file e-71-0o119-fig1.tif]

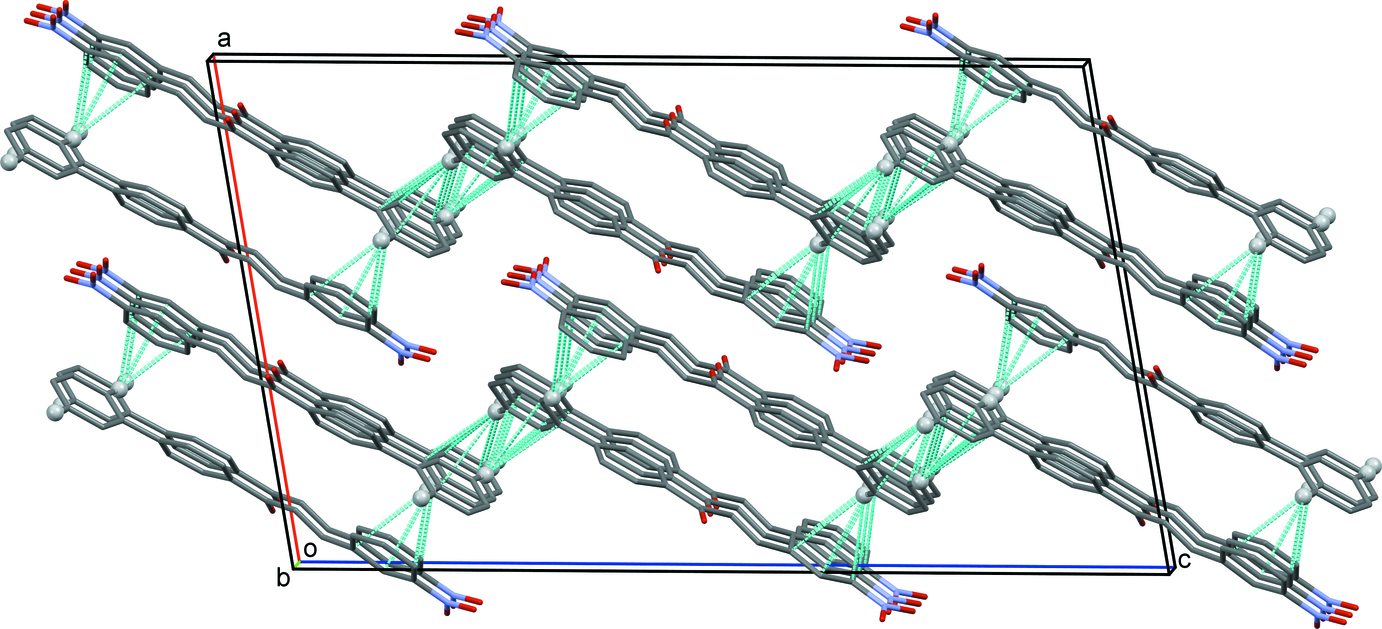

Supplement: Supplementary file 6 [file e-71-0o119-fig2.tif]
